# A Retrospective Study on the Significance of Liver Biopsy and Hepatitis B Surface Antigen in Chronic Hepatitis B Infection

**DOI:** 10.1097/MD.0000000000002503

**Published:** 2016-03-03

**Authors:** Da-Wu Zeng, Jie-Min Zhang, Yu-Rui Liu, Jing Dong, Jia-Ji Jiang, Yue-Yong Zhu

**Affiliations:** From Liver Center (D-WZ, Y-RL, JD, J-JJ, Y-YZ); and Department of Pharmacy, The First Affiliated Hospital, Fujian Medical University, Taijiang District, Fuzhou, Fujian, China (J-MZ).

## Abstract

To investigate changes in the HBV replication level along with the natural course of chronic HBV infection and to examine the accuracy of the immune tolerant phase defined by the serological profile.

A total of 390 chronic HBV-infected patients were retrospectively recruited for this study. They were classified into immune-tolerance (IT), immune-clearance (IC), low-replicative (LR), and HBeAg-negative hepatitis (ENH) phases according to serological profiles (single-standard, SS) or dual-standard (DS) with the inclusion of liver histology. Serum HBV DNA and HBsAg were quantitatively measured, and liver histology was quantitatively analyzed.

The accuracy of the SS-defined IT phase was low, and active pathological changes were detected in 56 of 112 SS-defined IT patients. DS-defined IT patients had higher HBsAg levels (*P* = 0.0002) than the SS-defined patients. The quantitative HBsAg level can help identify SS-defined IT patients with potential liver injury. The area under the received operating characteristic curve for predicting the DS-defined IT phase was 0.831 (HBsAg 4.398 log IU/mL; sensitivity 87.5%; specificity 73.2%). HBV DNA was reduced by 4 logs, whereas HBsAg was only decreased by 2 logs with HBeAg positive to negative phase conversion.

Approximately half of IT patients defined by SS may have medium or severe liver injury. Quantitative measurement of the HBsAg level can help identify SS-defined IT patients with potential liver injury.

## Abstract 2

Supplemental Digital Content is available in the text

## INTRODUCTION

Chronic hepatitis B virus (HBV) infection is a serious global health concern. Each year more than 1 million chronic HBV carriers worldwide die of liver cirrhosis and hepatocellular carcinoma, the most severe adverse sequelae of chronic HBV infection.^1,2^ It was estimated that approximately 6% to 20% of chronic HBV-infected patients will develop cirrhosis within 5 years if not treated, indicating the importance of mitigating liver injury. The current guidelines recommend initiation of anti-HBV therapy based on the natural course of chronic HBV infection, which consists of 4 phases: the immune-tolerance (IT), immune-clearance (IC), low-replicative (LR), and hepatitis B early antigen (HBeAg)-negative hepatitis (ENH) phases.^3–5^ Those phases are characterized by distinct patterns of serologic markers (HBeAg and hepatitis B surface antigen [HBsAg]), HBV DNA level, and level of serum alanine transaminase (ALT). HBsAg is the hallmark of HBV infection, and quantitative detection of HBsAg is available clinically.^6^ However, the correlation between quantitative HBsAg and HBV DNA level is controversial and may depend on HBV genotypes.^7–10^

Antiviral therapy is currently not recommended for IT phase patients, which is marked by high serum HBV DNA but normal ALT level. However, clinical evidence has shown that a proportion of patients at this phase may experience active liver injury, suggesting an antiviral therapy is required. Liver injury and histologic characteristics can be reliably revealed in liver biopsy specimens.^11,12^ However, current HBV management guidelines (APASL, EASL, and AASLD) do not recommend liver biopsy as a routine procedure for determining liver injury, but it should be considered only under certain circumstances^13^ due to its invasive nature.

In the present study, we studied the accuracy of the serological profile-defined IT phase, with help of liver histology, and investigated the role of quantitative HBsAg detection in identifying the IT phase patients with potential liver injury. We also compared the changes in serum HBeAg and HBV DNA levels in patients at different chronic infection phases using serological profile (single standard [SS]) or both serological and histological profiles (dual standard, DS). A portion of patients who received the antiviral treatment during the IT phase was followed longitudinally for a median duration of 14 months.

## MATERIALS AND METHODS

### Patients

This retrospective cohort study included a total of 390 treatment-naive patients (302 men and 88 women) who were chronically infected with HBV (positive for the HBsAg for at least 6 months). These patients were consecutively treated at the Liver Center of the First Affiliated Hospital of Fujian Medical University between January 2010 and April 2014. Four exclusion criteria outlined in a previous report were applied in this study.^14^ The study followed the principle of the World Medical Association Declaration of Helsinki and was approved by the Institutional Review Board of the First Affiliated Hospital, Fujian Medical University. Patients provided written consent voluntarily for their information to be stored in the hospital database and used for research.

### Quantification of Serum HBV DNA, HBsAg, and HBeAg Levels and HBV Genotyping

HBV DNA levels were measured with quantitative PCR assay (PG Company, Shenzhen, China). The test detection range is 500 to 1.0 × 10^9^ IU/mL. HBsAg was quantified using the Architect platform (Abbott Laboratories, Chicago, IL) per the manufacturer's instructions, and it was calibrated using the WHO standard for HBsAg. HBeAg levels were measured using the AxSYM microparticle enzyme immunoassay (Abbott). The AxSYM assay measures the ratio of the sample (S) to the cut-off (Co) (S/Co ratio), and an S/Co ratio ≥1.0 is defined as HBeAg-positive. Detection of HBV genotypes was performed as previously described.^15^ Other serum biochemical parameters were determined using a biochemistry analyzer within 1 week prior to liver biopsy.

### Liver Histology and Quantification of Fibrosis

A single liver biopsy was performed in all patients recruited in this study using a 16G Tru-Cut needle (TSK Laboratory, Tochigi-Ken, Japan) guided by color Doppler ultrasound (ACUSON, Aspen Advanced Ultrasound, Siemens Company, New Jersey). For most biopsy, more than 11 portal tracts of liver tissue specimens (maximum was 28 and minimum was 6) with a length of 15 to 20 mm were obtained and fixed in 4% neutral formalin before embedding in paraffin. All sections were stained with hematoxylin-eosin-safran (HE) and Masson trichrome. Liver histological activity was independently evaluated by at least 2 pathologists according to Histological Activity Index numerical scoring system. As described by the guidelines, clinical phases for each patient were defined using either the serological profile only (HBeAg, HBV DNA, and ALT; SS) or DS (serological + histological profiles) (Table 1).^16,17^ The IT phase (DS) was defined by HBsAg positivity for 6 months or more, HBeAg positive, HBV DNA > 1.0 × 10^5^ IU/mL, normal or minimally elevated ALT (<2 times ULN), and minimal or no histological changes on liver biopsy.^9,17–20^

**TABLE 1:**
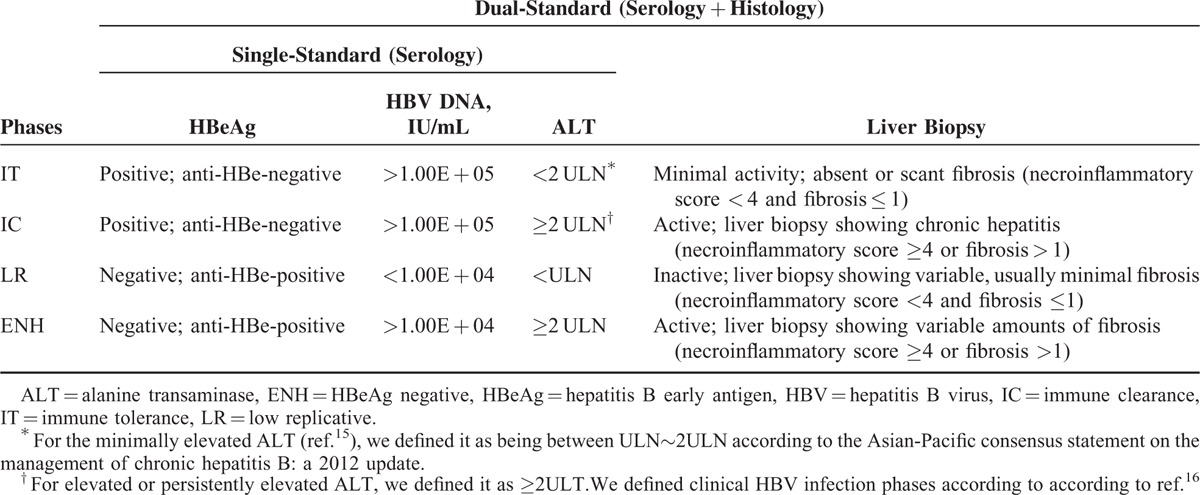
Criteria to Define Clinical HBV Infection Phases

### Statistical Analysis

Quantitative data are presented as means ± standard deviation (SD). Statistical analysis was performed using SPSS 16.0 software (SPSS Inc., Chicago, IL). Mann–Whitney test was used to compare the results for continuous and categorical variables between groups. Kruskall–Wallis analysis of variance (ANOVA) was used to compare nonparametric continuous data, and Fishers exact test was applied for comparing categorical data. Spearman correlation coefficients (*r*) were used to describe the correlations between 2 variables.

## RESULTS

### Clinical Characteristics of Patients During Different Phases as Determined by SS or DS

Of 390 chronic hepatitis B (CHB) patients, 220 were HBeAg positive and 170 were HBeAg negative (Supplementary Figure 1). The number of patients classified into different stages varied between SS and DS. A total of 142 patients did not fit a typical phase by the DS definition. Particularly, it is noteworthy that 56 HBeAg-positive patients (56/112, 50%) were excluded from the typical IT phase by the DS definition. Of these 56 patients, not all of them were willing to initiate the antiviral treatment and some treatment subjects were lost during the follow-up. A final 22 patients were treated with antivirals and were monitored by follow-up to 92 weeks. In addition, DS-defined IT patients had significantly lower levels of aspartate transaminase (28.9 ± 8.7 IU/L, *P* < 0.01) and gamma glutamyl transpeptidase (28.9 ± 21.2 IU/L, *P* < 0.05) than SS-defined IT patients (35.4 ± 15.7 and 40.5 ± 35.5 IU/L; Supplementary Table 1).

### Serum HBsAg, HBeAg, and HBV DNA Levels During Different Phases as Defined by SS or DS

**FIGURE 1:**
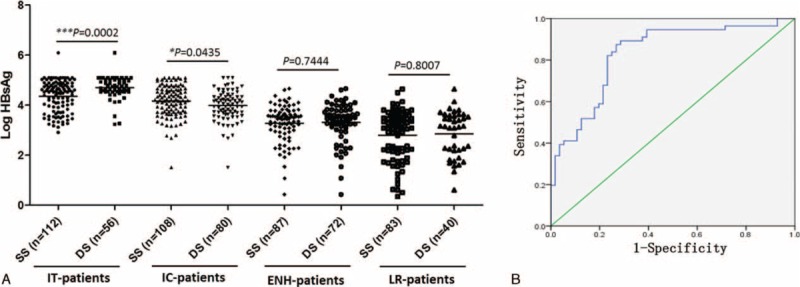
Comparison of HBsAg levels in CHB patients at different phases defined by SS or DS (A). ROC curve for HBsAg levels and liver activity (B). AUROC = area under the ROC curve, CHB = chronic hepatitis B, CI = confidence interval, DS =  = dual standard, ENH = HBeAg negative hepatitis, HBsAg = hepatitis B surface antigen, IC = immune-clearance, IT = immune-tolerance, LR = low-replicative, ROC = receiver operating characteristic, SS = single standard, Std = standard.

As shown in Table 2, the HBsAg level in DS-defined IT patients (n = 56; 4.704 ± 0.4578 log IU/mL (95% CI, 4.581–4.826) was significantly higher than that in SS-defined IT patients (n = 112; 4.354 ± 0.6179 log IU/mL, 95% CI 4.238–4.470; *P* = 0.0002; Figure 1A). In contrast, DS-defined IC patients (n = 80) had a lower HBsAg level (3.981 ± 0.6053 log IU/mL, 95% CI 3.846–4.116) than SS-defined IC patients (n = 108; 4.167 ± 0.6326 log IU/mL, 95% CI 4.046–4.288; *P* = 0.0435). No significant difference in HBsAg levels was found between ENH and LR patients defined by the 2 methods (Figure 1A). The HBsAg levels were reduced by approximately 1.3 to 1.5 logs from the IT/IC phases to the LR phase (4.354/4.167 to 2.803 log IU/mL by SS or 1.1 to 1.9 logs from 4.704/3.981 to 2.850 log IU/mLby DS; Table 2). IT patients were defined to be positive for HBeAg and negative for HBeAb, HBV DNA >1.00E + 05 IU/mL and ALT < 2 ULN (Table 1). To obtain a cut-off level of HBsAg that can predict the DS-defined IT phase probability (little or no liver injury on liver biopsy), we plotted a receiver operating characteristic (ROC) curve (Figure 1B) based on the log values of HBsAg and the extent of liver injury. We found that when the log value of HBsAg was 4.398, the area under the ROC curve (AUROC) of the prediction model for the DS-defined IT phase probability was 0.831 (sensitivity 87.5%, specificity 73.2%).

**TABLE 2:**
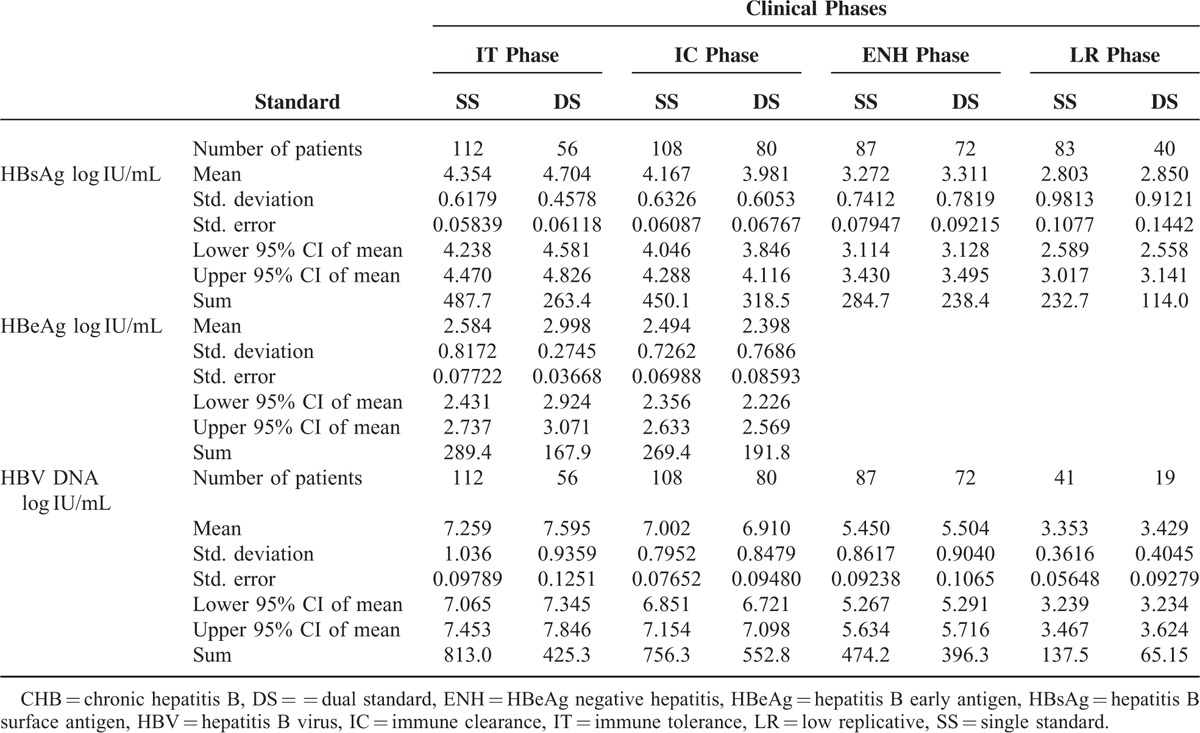
Serum Levels of HBsAg, HBeAg, and HBV DNA in CHB Patients at Different Phases Defined by 2 Standards

**FIGURE 2:**
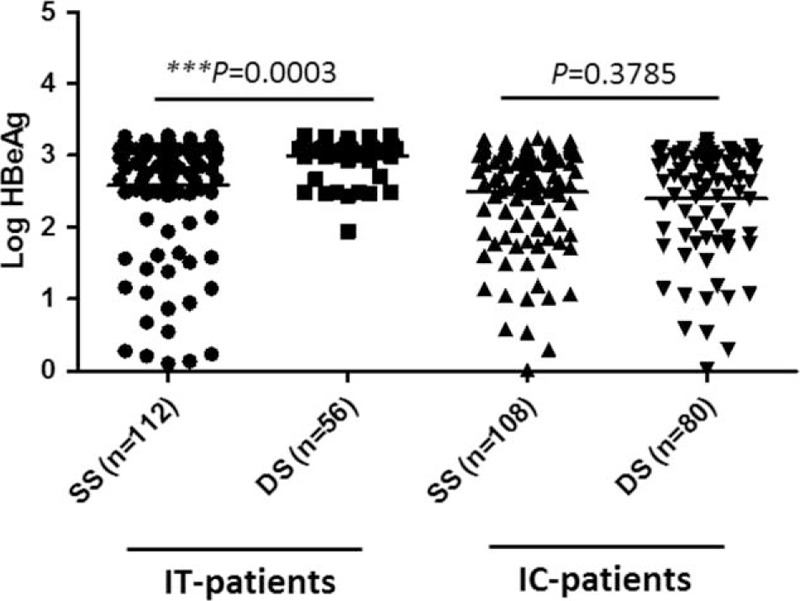
Comparison of HBeAg levels in CHB patients at the IT or IC phase defined by SS or DS. CHB = chronic hepatitis B, CI = confidence interval, DS =  = dual standard, ENH = HBeAg negative hepatitis, HBeAg = hepatitis B early antigen, IC = immune-clearance, IT = immune-tolerance, LR = low replicative, SS = single standard, Std = standard.

**FIGURE 3:**
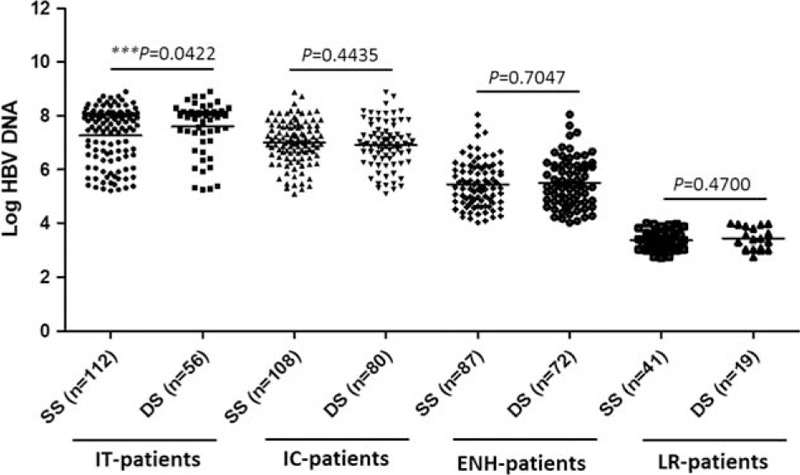
Comparison of HBV DNA levels in CHB patients at different phases defined by SS or DS. CHB = chronic hepatitis B, CI = confidence interval, DS =  = dual standard, ENH = HBeAg negative hepatitis, HBV = hepatitis B virus, IC = immune-clearance, IT = immune-tolerance, LR = low-replicative, SS = single standard, Std = standard.

The HBeAg level in DS-defined IT patients was significantly higher than in SS-defined IT patients (2.998 ± 0.2745 vs 2.584 ± 0.8172 log IU/mL, *P* = 0.0003; Table 2 and Figure 2). No significant difference in HBeAg levels was observed among IC patients defined by the 2 methods (Figure 2). Similarly, only DS-defined IT patients had significantly higher levels of HBV DNA than SS-defined IT patients (7.595 ± 0.9359 vs 7.259 ± 1.036 log IU/mL, *P* = 0.042; Table 2 and Figure 3). The HBV DNA level was decreased by nearly 4 logs after the seroconversion from HBeAg positive to negative (7.259/7.002 to 3.353 log IU/mL by SS or 7.595/6.910 to 3.429 log IU/mL by DS; Figure 3).

### Correlations Between HBsAg and HBV DNA Levels

**FIGURE 4:**
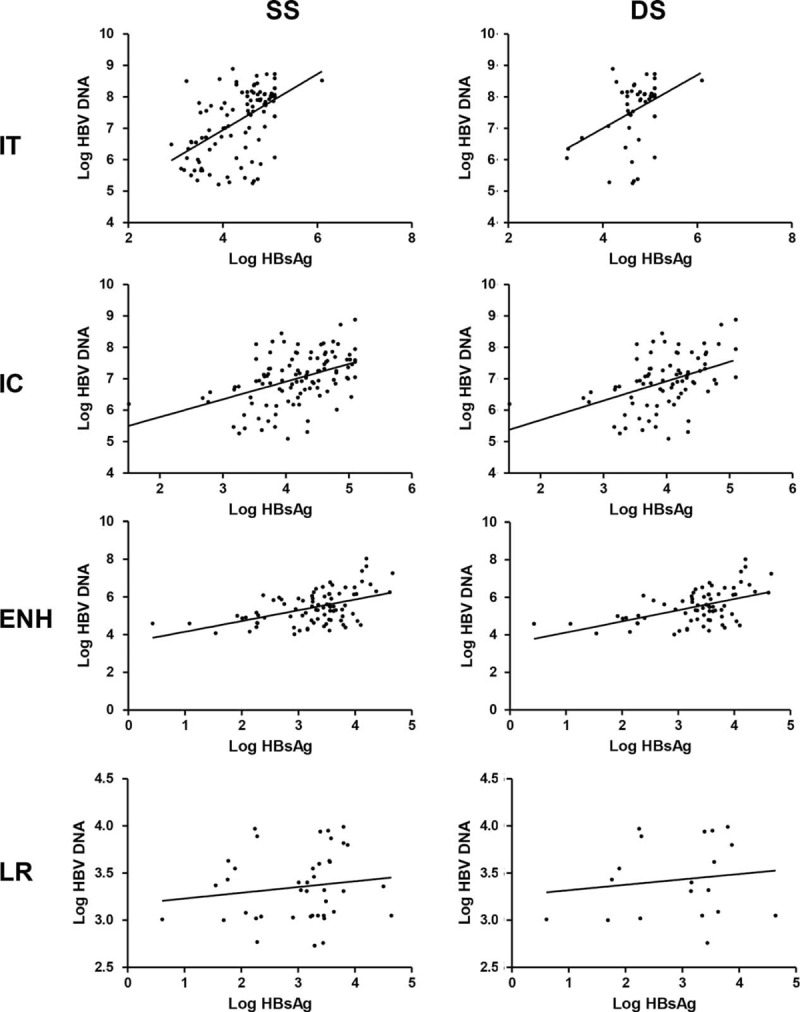
Correlation analysis of serum levels of HBsAg with HBV DNA in CHB patients at different phases defined by SS or DS. CHB = chronic hepatitis B, DS =  = dual standard, ENH = HBeAg negative hepatitis, HBsAg = hepatitis B surface antigen, HBV = hepatitis B virus, IC = immune clearance, IT = immune tolerance, LR = low replicative, SS = single standard.

Serum HBsAg levels positively correlated with HBV DNA levels in the IT, IC, and ENH phases defined by either SS (*r* = 0.5137, 0.4577, and 0.4574, all *P* < 0.0001) or DS (*r* = 0.3143, *P* = 0.0183; *r* = 0.4576, *P* < 0.0001; and *r* = 0.5031, *P* < 0.0001; Figure 4). No correlation between HBsAg and HBV DNA levels was observed in LR phase patients defined by the 2 methods (Figure 4). The HBV genotypes of patients enrolled in this study were HBV genotype B or C. There was no difference in the levels of HBsAg or HBV DNA between genotype B and C patients.

### Follow-Up of 22 Patients Who Were Excluded From the IT Phase by DS and Received Antiviral Therapy

Based on the liver biopsy findings, 142 patients were excluded because they did not fit within a typical phase of chronic HBV infection. Among these patients (Supplemental Table 2), 22 SS-defined IT patients who were experiencing active liver injury detected in the sections were subjected to antiviral treatment (Histological Activity Index ≥4 or at least grade A2 or stage F2, indicative of moderate to severe active necroinflammation and/or fibrosis).^21–25^ During the follow-up, 9 of 20 (45%) lost detectable HBV DNA levels within 6 months and 19 of 22 (86.4%) within 36 months. Three cases had HBeAg seroconversion at 48 weeks and another 3 cases at 92 weeks. The total percentage of HBeAg seroconversion was 33.3% (6 out of 18).

## DISCUSSION

The purposes of this study were to determine changes in the HBsAg level along the natural course of chronic HBV infection and to investigate the accuracy of defining the IT phase using the serological profile alone, comparing the classification with dual profiles of serology and histology. One of main findings from this cohort was that the accuracy of diagnosing the IT phase using the SS was low, and only 56 of 112 IT phase patients identified by SS were confirmed by DS. The primary reason for such low accuracy with SS is that ALT was normal, and it was not sensitive enough to identify those patients who had active liver injury. The percentage of IT phase patients with active injury in this study was similar to published data. Thus, approximately 50% of IT patients may not have been given antiviral treatment based on the SS definition. Consequently, liver disease in those patients who required antiviral treatment but were not treated may have progressed.

Indeed, the so-called IT phase that characterizes the early phase of chronic HBV infection has now been challenged by increasing clinical and epidemiological data. The absence of biochemical markers of liver inflammation does not signal the absence of an HBV-specific T-cell response.^26^ The immunopathological events during chronic HBV infection are likely associated with age-dependent changes, as older patients have stronger immunity.^27^ It has been reported that HBeAg-positive subjects older than 40 years with persistently “high normal” ALT levels may have significant hepatic necroinflammation or fibrosis.^28^ Conversely, CHB patients in the IT phase are usually young,^29^ but most of the CHB patients enrolled in our study were adults. Therefore, liver inflammation in IT patients may not as common as observed in our study. However, regardless of patients’ age, we showed that HBeAg-positive CHB patients with HBsAg > 4.398 log IU/mL but a normal to minimally elevated ALT may have significant potential for liver injury. The area under the ROC curve (AUROC) of the prediction model for the DS-defined IT phase probability was 0.831 (sensitivity 87.5%, specificity 73.2%). Our results, together with published data, highlighted the challenge in managing those IT phase patients. One solution to this problem is to selectively perform liver biopsy in those patients in whom active liver injury is suspected. It appeared that the serum HBsAg level in our cohort could be used as an initial indicator for IT patients with active liver injury. We also revealed that the HBsAg levels were increased in IT patients whereas they were decreased in IC patients (*P* < 0.05) in the DS-defined patients. Thus, we propose that the SS-defined IT phase patients should be further evaluated via quantitative HBsAg detection. The patient should be considered for liver biopsy if the serum HBsAg level is lower than 4.398, an indicator for potential liver injury. We hope that the inclusion of quantitative HBsAg measurement for identifying patients with potential liver injury for liver biopsy will minimize the number of IT patients with liver injury who are mistakenly considered healthy.

The HBV DNA level is steadily reduced, as is the HBeAg level as the course of chronic HBV infection progresses. As our results showed, the HBV DNA level was decreased by nearly 4 logs after the seroconversion from HBeAg positive to negative. However, the HBsAg level was reduced by only 2 logs during the same period. Our results suggest that the host is strengthening the antiviral restriction on HBV replication along with the course of chronic HBV infection. This restriction leads to 2 outcomes: 1st, HBV covalently closed circular DNA (cccDNA) in the infected liver is significantly reduced as reflected by reduced HBV DNA and HBsAg levels and HBeAg seroconversion. This understanding is consistent with earlier reports, which found that the cccDNA was reduced by 100-fold along with HBeAg seroconversion,^30^ and the reduced intrahepatic cccDNA may imply more effective control of HBV infection by the host immunity.^31^ A new strategy exploiting lymphotoxin-b receptor activation has recently been shown to specifically and nonhepatotoxically degrade this nuclear form of HBV DNA.^32^ Second, there is selective inhibition of pregenomic RNA (pgRNA) transcription, leading to reduced HBeAg synthesis and HBV DNA replication. However, the transcription of S gene remains active, leading to a relatively higher HBsAg level. Such selective inhibition of viral gene transcription can be verified by studying the copy number of pgRNA and S RNA molecules from the liver. This will shed new light on understanding why the HBsAg level is still high in the late phase of chronic HBV infection if our proposed mechanisms are verified.

Long-term follow-up of 22 patients who were excluded from the IT-phase by DS and received antiviral treatment showed the significant inhibition of viral replication, leading to HBeAg seroconversion (6 of 18, 33.3% after 36 months) and undetectable HBV DNA (19 of 22, 86.4% within 36 months). These results indicate that the inclusion of liver biopsy may make an important difference in treatment decisions for IT patients who do not meet the current guidelines.^33^

There were several limitations in this study. It was a retrospective cross-sectional study, and we did not have the kinetics data overtime. The kinetics changes in virological and serological markers would enable us to better understand the nature of chronic HBV infection. Second, as the study participants were patients who visited our department for liver-related illness, the proportion of patients with liver injury in the IT phase may be overestimated in this study. In addition, our study did not consider the impact of the patients’ age, although IT patients are usually young and would be at less risk for developing hepatic inflammation. We will include IT patients who do not feel need to visit their hepatologists in a future multicenter study and use age as a covariant factor. Last, we did not have liver samples to analyze HBV markers including cccDNA, rcDNA, and viral RNAs in infected cells. The detection of intrahepatic markers could illustrate the details of the host's restriction of viral replication along the time course.

In summary, the accuracy of the SS-defined IT phase was low, and only 50% of the identified patients were in the true IT phase as verified by histology. The inclusion of quantitative HBsAg measurement could further identify the SS-defined IT patients who may have active liver injury with normal ALT, for liver biopsy. Our results suggest that HBV DNA replication was increasingly inhibited along with HBeAg seroconversion. This inhibition likely occurs at both the cccDNA and transcription levels. It appears that a selectively stronger inhibition was placed on the pgRNA than on S gene transcription.

## Supplementary Material

Supplemental Digital Content
