# Supplementary material for: A Retrospective Study on the Significance of Liver Biopsy and Hepatitis B Surface Antigen in Chronic Hepatitis B Infection
Source: Medicine (Baltimore). 2016 Mar 3;95(8):e2503. doi: 10.1097/MD.0000000000002503 (PMC4778991; doi:10.1097/MD.0000000000002503)
Supplement: Supplemental Digital Content [file medi-95-e2503-s001.doc]

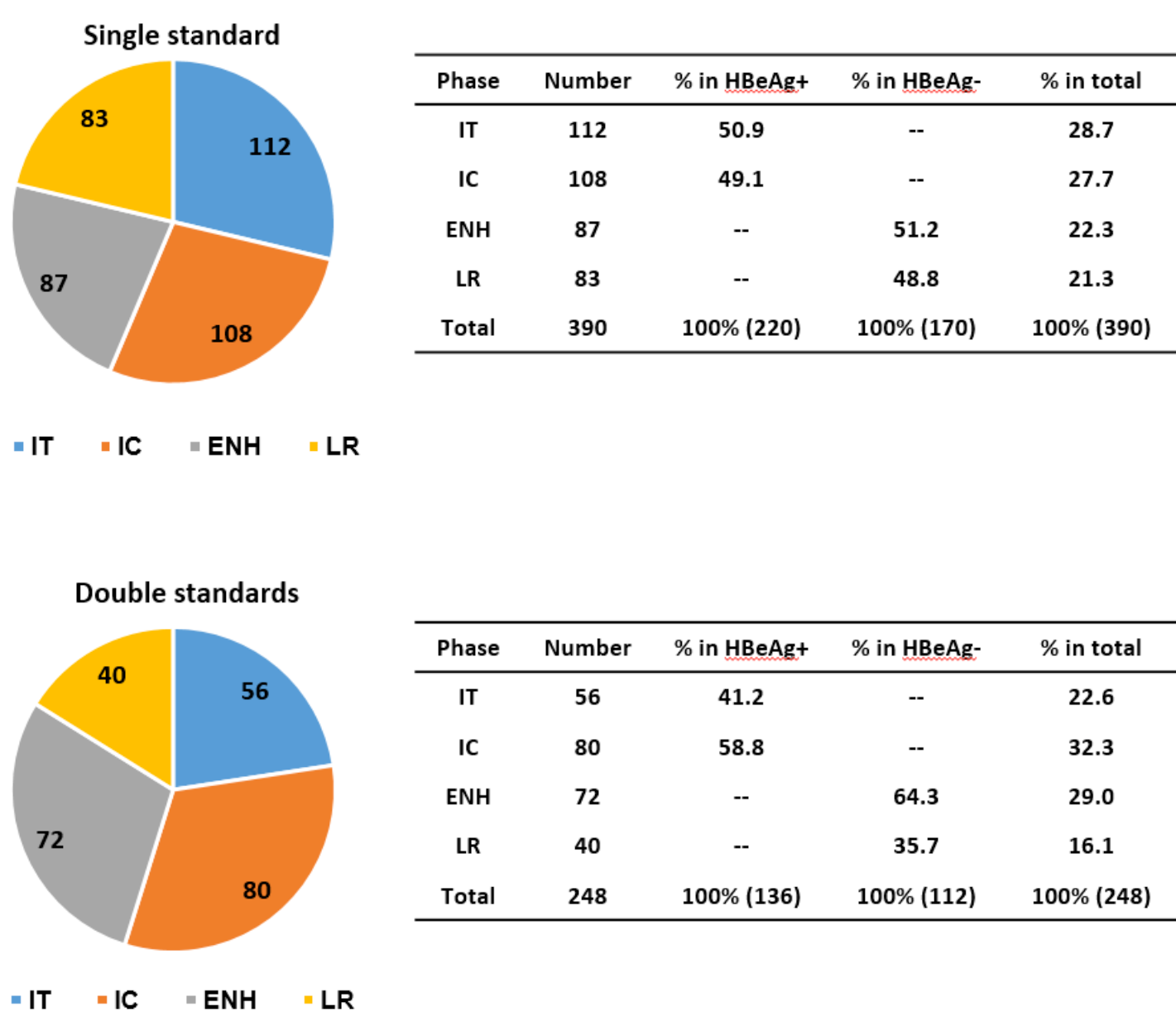


**Supplemental Table 1. Clinical characteristics of the patients in different phases categorized by the two methods**

| **Characteristics** | **Clinical phases** | | | | | | | |
| --- | --- | --- | --- | --- | --- | --- | --- | --- |
|  | **IT phase** | | **IC phase** | | **ENH phase** | | **LR phase** | |
|  | **SS^#^**  **(n=112)** | **DS^&^**  **(n=56)** | **SS**  **(n=108)** | **DS**  **(n=80)** | **SS**  **(n=87)** | **DS**  **(n=72)** | **SS**  **(n=83)** | **DS**  **(n=40)** |
| **Age, yrs** | 36.4 ± 10.0^##^ | 35.9 ± 9.3 | 32.7 ± 10.5 | 34.2 ± 10.5 | 38.7 ± 10.7 | 38.1 ± 10.5 | 39.8 ± 8.3 | 38.9 ± 7.5 |
| **Male:female** | 82:30 | 44:12 | 84:24 | 61:19 | 70:17 | 58:14 | 66:17 | 33:7 |
| **TBil, µmol/L** | 12.6 ± 5.5 | 13.3 ± 6.1 | 20.9 ± 17.4 | 21.5 ± 18.3 | 26.1 ± 27.8 | 28.2 ± 30.0 | 15.4 ± 6.6 | 15.3 ± 6.5 |
| **ALB, g/L** | 41.1 ± 5.1 | 42.1 ± 5.9 | 40.8 ± 4.8 | 40.1 ± 3.9 | 40.1 ± 5.3 | 39.5 ± 5.3 | 43.2 ± 5.0 | 45.2 ± 3.7* |
| **GLO, g/L** | 28.4 ± 4.9 | 27.4 ± 4.5 | 29.8 ± 4.3 | 30.0 ± 4.3 | 30.4 ± 4.9 | 30.8 ± 5.1 | 27.8 ± 4.3 | 27.1 ± 4.1 |
| **ALT, IU/L** | 42.8 ± 16.8 | 38.5 ± 16.4 | 314.5 ± 310.4 | 333.4 ± 335.0 | 302.8 ± 267.0 | 314.2 ± 241.8 | 27.3 ± 11.0 | 28.0 ± 12.8 |
| **AST, IU/L** | 35.4 ± 15.7 | 28.9 ± 8.7** | 180.6 ± 195.5 | 198.6 ± 215.7 | 159.4 ± 142.3 | 171.9 ± 147.6 | 25.8 ± 7.1 | 24.4 ± 5.6 |
| **GGT, IU/L** | 40.5 ± 35.5 | 28.9 ± 21.2* | 93.6 ± 82.2 | 106.8 ± 88.2 | 91.6 ± 65.1 | 99.6 ± 66.0 | 41.8 ± 64.0 | 30.2 ± 20.2 |
| **CHE, IU/L** | 8053 ± 2405 | 8643 ± 2500 | 7048 ± 2338 | 6652 ± 2285 | 7724 ± 3824 | 7460 ± 3989 | 8550 ± 2624 | 9610 ± 1831* |
| **INR** | 1.04 ± 0.14 | 1.01 ± 0.09 | 1.05 ± 0.12 | 1.07 ± 0.12 | 1.06 ± 0.12 | 1.06 ± 0.12 | 1.01 ± 0.09 | 0.98 ± 0.08 |
| **PLT, 10^11^/L** | 201.9 ± 54.9 | 215.9 ± 53.2 | 189.4 ± 58.4 | 179.1 ± 54.6 | 182.7 ± 53.0 | 180.2 ± 54.1 | 193.0 ± 63.9 | 211.1 ± 47.0 |

^#^SS, single standard

^&^ DS, double standard

^##^ Data are expressed as mean ± standard deviation (SD)

^*^ p<0.05

^**^ p<0.01

Other abbreviations: IT-immune tolerance; IC-immune clearance; ENH-HBeAg negative; LR-low replicative; TBil: total bilirubin; ALB: albumin; GLO: globulin; ALT: alanine aminotransferase; AST: aspartate aminotransferase; GGT: gamma glutamy ltranspeptidase; CHE: total cholesterol; INR: international normalized ratio; PLT: platelet

Supplemental Table 2. Hepatitis e antigen and HBV DNA levels in IT-excluded patients by the double standard during long-term follow-up

| **Patient** | **Baseline** | |  | **3–6 months**^#^ | |  | **9–12 months** | |  | **15–36 months** | |
| --- | --- | --- | --- | --- | --- | --- | --- | --- | --- | --- | --- |
|  | **HBeAg*** | **HBV DNA** |  | **HBeAg** | **HBV DNA** |  | **HBeAg** | **HBV DNA** |  | **HBeAg** | **HBV DNA** |
| 1 | 113 | 8.07E+07 |  |  | 500^&^ |  |  |  |  | **4** | **500** |
| 2 | 1 | 4.53E+05 |  |  | 500 |  | 2 | 500 |  | 0 |  |
| 3 | 40 | 2.20E+05 |  | 31 | 989 |  | 32 | 500 |  | 29 | 500 |
| 4 | 891 | 3.66E+07 |  | 485 | 1700 |  | 354 | 2290 |  |  |  |
| 5 | 1059 | 5.41E+07 |  | 676 | 1.41E+06 |  | 343 | 6830 |  |  |  |
| 6 | 7 | 3.13E+07 |  | 4 | 500 |  | 5 | 500 |  | 4 | 500 |
| 7 | 961 | 2.44E+08 |  | 671 | 6.75E+05 |  | 923 | 6.84E+06 |  |  |  |
| 8 | 1018 | 7.79E+07 |  | 310 | 500 |  | 248 | 500 |  |  |  |
| 9 | 577 | 5.31E+06 |  | 750 | 711 |  | 979 | 500 |  | 563 | 500 |
| 10 | 38 | 6.43E+07 |  | 20 | 506^$^ |  | 14 | 694 |  | 10 | 500 |
| 11 | 567 | 1.00E+06 |  | 1432 | 500 |  | 1771 | 500 |  | 0 | 500 |
| 12 | 336 | 3.69E+08 |  |  |  |  | 11 | 500 |  | 8 | 500 |
| 13 | 2 | 4.60E+05 |  |  |  |  | 1 | 500 |  |  | 500 |
| 14 | 1 | 4.64E+05 |  | 0 | 500 |  |  | 500 |  |  | 500 |
| 15 | 1334 | 8.03E+07 |  | 0 | 780 |  | 0 | 500 |  |  |  |
| 16 | 32 | 4.58E+05 |  | 10 | 519^$^ |  | 11 | 500 |  | 6 | 500 |
| 17 | 670 | 3.58E+06 |  | 100 | 1100 |  | 24 | 500 |  | 0 | 500 |
| 18 | 26 | 3.18E+08 |  |  |  |  |  |  |  | 4 | 500 |
| 19 | 139 | 3.79E+06 |  |  |  |  |  | 500 |  |  | 500 |
| 20 | 338 | 5.25E+07 |  |  | 770 |  |  | 500 |  |  |  |
| 21 | 519 | 3.36E+07 |  | 114 | 3.79E+04 |  |  | 500 |  |  |  |
| 22 | 24 | 2.10E+06 |  | 8 | 500 |  |  | 500 |  |  | 500 |

* HBeAg: S/Co

^&^500 was the lowest detection limit for HBV DNA and was considered as HBV DNA-negative.

^$^ Due to the sensitivity of the assay, 506 and 519 were also considered as negative.

^#^ Measured within 6 months. If multiple measurements were available during this period, the last one was counted. A similar rule was applied to the definition of 9–12 months and 15–36 months.
